# Beta cell regeneration upon magainin and growth hormone treatment as a possible alternative to insulin therapy

**DOI:** 10.1002/2211-5463.13556

**Published:** 2023-01-25

**Authors:** Azam Moosavi, Razieh Yazdanparast

**Affiliations:** ^1^ Institute of Biochemistry and Biophysics University of Tehran Iran

**Keywords:** differentiation, GABA, growth hormone, Magainin‐II, trans differentiation, β‐like cells

## Abstract

Insulin therapy, pancreas transplantation and β cell regeneration are among the suggested treatment strategies for type 1 diabetes. It has been shown that some antimicrobial peptides have the potential to increase insulin release and to improve glucose tolerance, although the mechanism by which they promote the regeneration of damaged pancreatic cells to functional β‐like cells remains unknown. To answer this question, we evaluated the *in vivo* effects of magainin‐AM2 and growth hormone (GH) on the regeneration of streptozotocin (STZ)‐damaged mouse pancreas. Treatment with magainin‐AM2 and GH ameliorated the effects of STZ on fasting blood glucose and glucose tolerance test values, and also resulted in a significant increase in total cell counts (α and β) and the number of insulin^+^ and glucagon^+^ cells per islet and a decrease in the number of T and B cells. In addition, we observed a 1.43‐ and 2.21‐fold increase in expression of paired box 4, one of the main factors for α to β‐like cell conversion, in normal‐ and diabetes‐treated mice, respectively. Similarly, expression of P‐S6 and extracellular signal‐regulated kinases 1 and 2, required for cell proliferation/differentiation, increased by 3.27‐ and 2.19‐fold among the diabetes‐treated and control diabetic mice, respectively. Furthermore, in all experiments, amelioration of the effects of STZ were greatest upon Mag treatment followed by GH administration. The present *in vivo* data provide evidence in support of the possibility of pharmaceutical induction of α cell production and their trans‐differentiation to functional β‐like cells.

## Graphical abstract

In the present study, normal and diabetic mice were treated with magainin, growth hormone or both. Theresults obtained, along with the observed improvement of fasting blood glucose and glucose tolerance test vallues, demonstrated compensation of β cells via induction of α/β cell production and their trans‐differentiation to functional β‐like cells. The best improvement was observed in the group treated with both magainin (Mag) and growth hormone (GH).

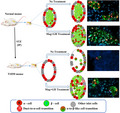

**Abbreviations**
ANOVA: analysis of variance
ARX: aristaless related homeobox
ERK: extracellular signal‐regulated kinase
FBS: fasting blood glucose
GH: growth hormone
GLP‐1: glucagon‐like peptide 1
GTT: glucose tolerance test
IHC: immunohistochemistry
Mag: magainin
PAX4: paired box 4
PBS: phosphate‐buffered saline
STAT: signal transducer and activator of transcription
STZ: streptozotocin

Diabetes treatment is currently a universal challenge. In type 1 diabetes mellitus, the progressive loss of functional β cell mass occurs. Therefore, one main goal in the treatment of affected individuals is based on compensation of the endogenous insulin pool size via expanding the functional β cell population [1].

Toward this goal, the maturation of various cell sources capable of differentiation, dedifferentiation and trans‐differentiation has been suggested. Many transcription factors play roles in these events such as pancreatic and duodenal homeobox 1 [2], neurogenin 3 [3], aristaless related homeobox (ARX) and paired box 4 (PAX4) [4]. Among them, ARX and PAX4 have received more attention because of their roles in the final differentiation step of β cells. Studies have also shown that ARX inactivation in pancreatic glucagon^+^ cells can transform them into β‐like cells [5].

Among the antidiabetic agents under investigation, antimicrobial peptides have received extensive attention [6, 7]. Magainin‐AM2 (Mag II), as an orthologue of magainin‐2 from amphibians, is a cationic peptide with the lowest hemolytic activity, assumed to work via cell membrane depolarization, increasing intracellular calcium [6, 8] and enhancing the release of Glucagon‐like peptide 1 (GLP‐1) from GLUTag cells, followed by insulin‐release from the treated cells [9, 10]. Furthermore, Yazdanparast *et al*. [11] have reported the significant roles of Mag II on enhancing the mouse hypothalamic GABA content. Recently, Collombat *et al*. [12] have also shown that sustain use of GABA induces α to ß‐like functional cells. Regarding such information, we were interested in determining whether the endogenously‐induced GABA, via Mag modulation of the relevant main signaling elements, could bring about β cell regeneration. Our goal was supported by considering the fact that the majority of Mag investigations have been performed on type 2 diabetes mellitus with no reports on their roles in β cell regeneration. On the other hand, regarding the overlap among the signaling pathways of Mags and growth hormone (GH), as shown in Fig. 1 and Fig. S1, it would be interesting and beneficial to evaluate their combined influence on the regeneration of pancreatic β cells.

**Fig. 1:**
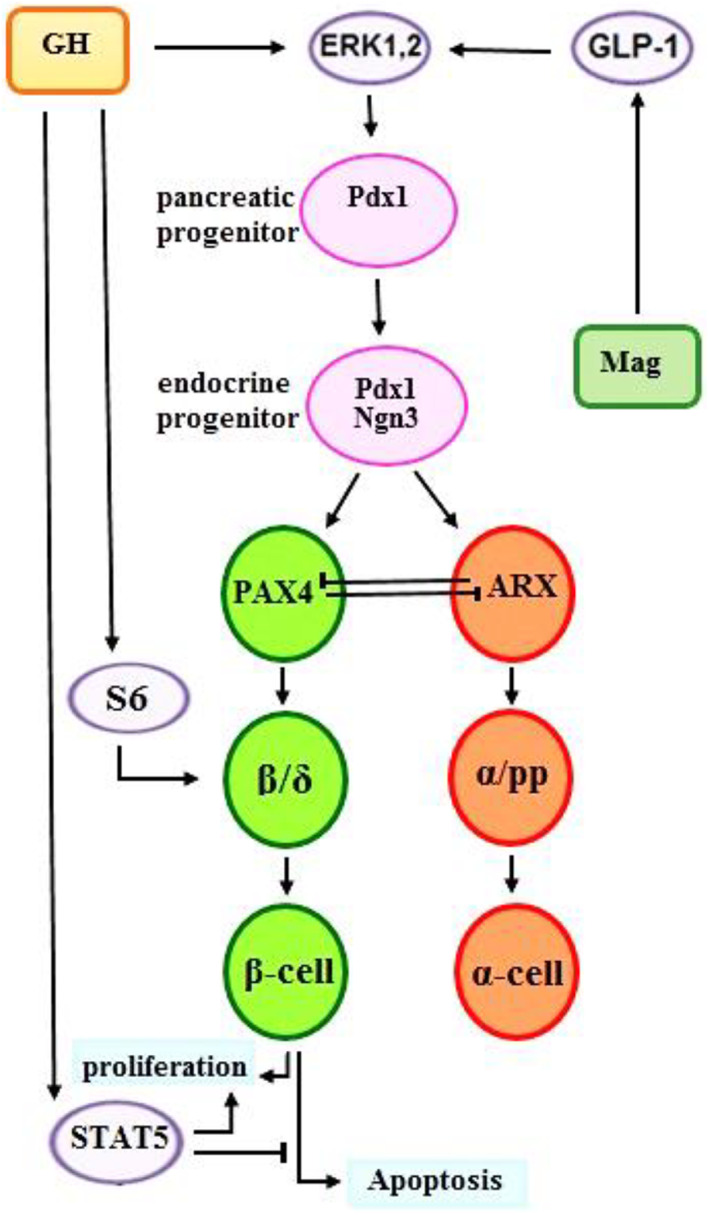
Key signaling elements under the influence of GH and/or Mag based on the literature [6, 21, 22].

Our data clearly indicated that the simultaneous use of Mag then GH enhanced more effectively the number of mice pancreatic β cells compared to those of mice treated only with Mag or GH.

## Materials and methods

### Study design

Type 1 diabetes mellitus was induced via i.p. injection of Balb/C male mice (weighing approximately 27–32 g) with multiple low doses of streptozotocin (STZ) (40 mg·kg^−1^) and the control group received an equal volume of the vehicle [13]. All mice were kept under standard conditions (at a controlled temperature of 21 ± 2 °C, under a 12 : 12 h light/dark photocycle, with *ad libitum* access to food and water) in the animal house, five per cage. The normal (N) (*n* = 20) and diabetic (D) (*n* = 20) groups were each divided into four subgroups (*n* = 5) for i.p. injections. The N1 and D1 received 6.7 mg·kg^−1^·day^−1^ GH for 14 days. The N2 and D2 received 0.185 mg·kg^−1^ Mag II daily for 28 days. The N3 and D3 first received 0.185 mg·kg^−1^ Mag II for 28 days followed by 6.7 mg·kg^−1^ GH for 14 days. The N4 and D4 received equal volume of physiological saline.Fasting bloood glucose (FBS) measurements were achieved before the treatments and weekly during the treatments. The glucose tolerance test (GTT) tests were performed at the end of each treatment. The pancreatic tissue of each mouse was quickly removed and placed in cold phosphate‐buffered saline (PBS). Pancreatic samples were collected for both extraction and storage at −80 °C and the remaining immersed in 4% paraformaldehyde for further investigation. Also hematoxylin and eosin staining and immunohistochemistry (IHC) were conducted as described previously [14]. All protocols especially animal maintenance and manipulation were conducted according to the guidelines of animal ethics committee of University of Tehran with approval ID of IR.UT.SCIENCE.1401.005.

### FBS/GTT measurements

The FBS levels were measured after fasting the mice for 6 h using an Accu‐Chek glucometer (Roche, Basel, Switzerland) via the tail vein. After the last day of treatment, FBS measurements were followed by a glucose injection (2 g·kg^−1^, i.p.). Then, at the indicated times, blood glucose levels were evaluated with an Accu‐Chek glucometer via the tail vein.

### Pancreatic protein extraction and western blotting

Pancreatic protein was extracted using a previously published protocol [15]. Each pancreatic tissue was extracted in the buffer containing Tris‐HCl (100 mm, pH 7.5), EDTA (10 mm), sodium pyrophosphate (10 mm), sodium fluoride (0.1 mm), sodium orthovanadate (10 mm), phenylmethanesulfonyl fluoride (2 mm) and aprotinin (10 mg·mL^−1^). Each homogenized pancreatic tissue was vortexed for 30 min at 4 °C. The homogenates were centrifuged at 10500 ***g*** for 20 min at 4 °C. The protein content of each supernatant was determined by Lowry's method [16] with fetal bovine serum as the calibrating agent. Each pancreatic extract was divided into aliquots and stored at −80 °C for future use.

For western blots, an aliquot of each pancreatic extract was added to the loading buffer (5 × loading buffer: 3 mL of 20% SDS added to 3.75 mL of 1 m Tris buffer at pH 6.8, 9 mg of bromophenol blue, 1.16 g dithiothreitol and 4.5 mL of glycerol, made up to a final volume of 15 mL with dH_2_0) and boiled for 5 min at 95 °C and then immersed in ice. Following SDS/PAGE, the resolved proteins were transferred to the poly(vinylidene difluoride) blotting membrane. The poly(vinylidene difluoride) membrane was then non‐specifically blocked with fat free milk and, after overnight incubation at 4 °C with primary antibody, horseradish peroxidase‐conjugated secondary antibody was added and incubated with gentle mixing for 120 min at room temperature. Finally, the images were developed based on a western blotting–electrochemiluminescence protocol [17].

### Hematoxylin and eosin staining and immunohistochemistry assay

Hematoxylin abd eosin staining of each fixed 6‐μm pancreatic section was achieved as described previously [14]. After embedding in paraffin, each tissue fixed in 4% paraformaldehyde was cut into 6‐μm sections and applied to slides. For hematoxylin and eosin staining, tissues were subjected to rehydration, incubation in hematoxylin (2.5 min), rinsing with water, dipping in 0.5% HCl/70% ethanol (v/v), rinsing with water, immersion in 0.2% NaHCO_3_, rinsing in water, dipping in 0.1% eosin for 20 s, rinsing briefly with water and, finally, dehydration and mounting.

For IHC evaluations, after embedding in paraffin, each tissue fixed in 4% paraformaldehyde was cut into 6‐μm sections and applied to slides. The sections were deparaffinized, rehydrated and then permeabilized in 0.2% Triton X‐100 for 5 min. Then, the permeabilized sections were blocked in PBS containing 10% inactivated fetal bovine serum for 90 min. Primary antibodies were provided in their required dilution in the same medium, applied on sections and then incubated overnight at 4 °C. After overnight incubation, each slide was incubated for 90 min with the appropriate secondary antibody after washing in PBS. Secondary antibodies were diluted in PBS containing 10% inactivated fetal bovine serum. Slides were viewed by fluorescence microscopy after washing in PBS and mounting with 4′,6‐diamidino‐2‐phenylindole [14].

The primary antibodies, including anti‐p‐extracellular signal‐regulated kinase (ERK)1/2 antibody sc‐81492 (dilution 1 : 1000), goat anti‐PAX4 antibody (ab101721) (dilution 1 : 1000), rabbit anti‐signal transducer and activator of transcription (STAT)5a antibody (ab30648) (dilution 1 : 1000), rabbit anti‐GAPDH antibody (ab181602) (dilution 1 : 1000), rabbit anti‐insulin antibody (ab63820) (dilution 1 : 500), mouse anti‐glucagon antibody sc‐514592 (dilution 1 : 500), mouse anti‐vimentin antibody sc‐6260 (dilution 1 : 500), rabbit recombinant anti‐Ki67 (ab197547) (dilution 1 : 500), rat anti‐CD3 antibody (ab11089) (dilution 1 : 500), mouse anti‐CD19 antibody (sc‐373897) (dilution 1 : 500), were used in western blotting and IHC assays. Also, all secondary antibodies were utilized at a concentration of 1 : 1000, including goat anti‐rat IgG H&L (ab6840), goat anti‐rabbit IgG H&L (ab6717), goat anti‐rabbit IgG H&L (ab72465), goat anti‐mouse IgG H&L (ab6785) and goat anti‐mouse IgG H&L (ab6787).

### Statistical analysis

All values are depicted as the mean ± SEM. *P* < 0.05 was considered statistically significant. The arbitrary optical density unit was acquired using imagej, version 1.46 (NIH, Bethesda, MD, USA). Data were analyzed using prism (GraphPad Software Inc., San Diego, CA, USA) by determining whether they followed a normal distribution using a D'Agostino‐Pearson omnibus normality test. If not, an unpaired/non‐parametric Mann–Whitney test was used. If positive, an unpaired *t*‐test (two groups being compared) or unpaired analysis of variance (ANOVA) (several groups compared simultaneously) was used assuming Gaussian distribution.

## Results

### Influence of Mag and GH on mice FBS and GTT

**Fig. 2:**
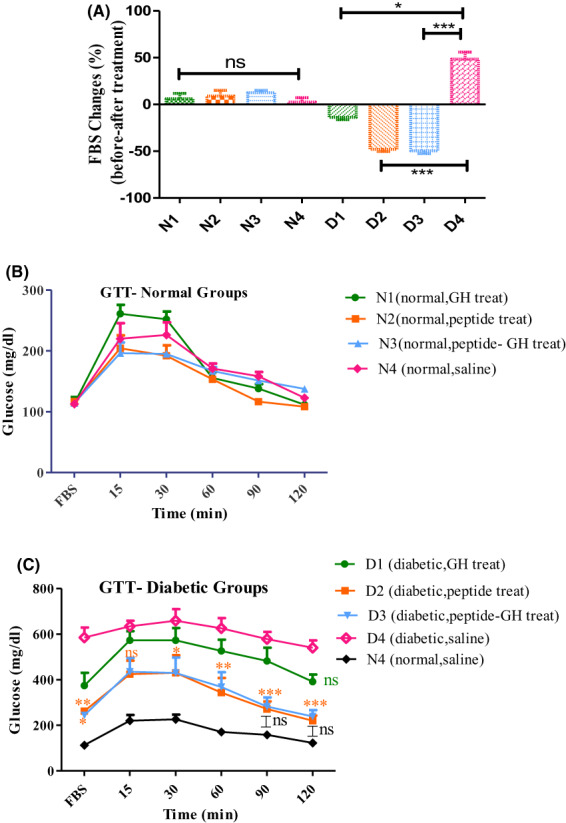
Comparative results of FBS and GTT among diabetic‐ and normal‐treated mice relative to their relevant controls. Comparison of the mean percentage of fasting blood glucose changes (A), the mean blood glucose at GTT defined intervals among treated‐ and untreated‐normal (B) and diabetic (C) mice. ***P < 0.001, **P < 0.01, *P < 0.05; ns, no significant (ANOVA). All data are depicted as the mean ± SEM (*n* = 5). N1 (normal, GH treated), N2 (normal, Mag treated), N3 (normal, Mag – GH treated), N4 (normal, saline treated), D1 (diabetic, GH treated), D2 (diabetic, Mag treated), D3 (diabetic, Mag + GH treated), D4 (diabetic, saline treated).

The slight increase in the mean percentage of FBS changes for normal mice (Fig. 2A) is not significant. By contrast, significant changes were observed for diabetic groups. In D4 diabetic mice, the mean percentage of FBS changes increased by 49.2%, whereas those of D1, D2 and D3 mice decreased by 8.2%, 47.9% and 49.6%, respectively. In other words, the approximate improvements in the fasting blood sugar for treated diabetic groups compared to untreated control diabetic ones were 57.4%, 97.1% and 98.8%. Figure 2B shows that the rise in blood sugar occurs in the first 30 min following glucose injection and this levels off to that of untreated normal ones at 120 min. This is in contrast to the rise of blood glucose in 15 min for the D1, D2 and D3 groups (Fig. 2C). Also, blood sugar levels for the untreated diabetic mice were higher than other groups at all defined times. As is evident from Fig. 2C, the blood glucose levels of D2 and D3 groups, in the first 90 and 120 min following glucose injection, had no significant difference with N4 (normal, untreated group).

### Evaluation of islet regeneration in diabetic mice

**Fig. 3:**
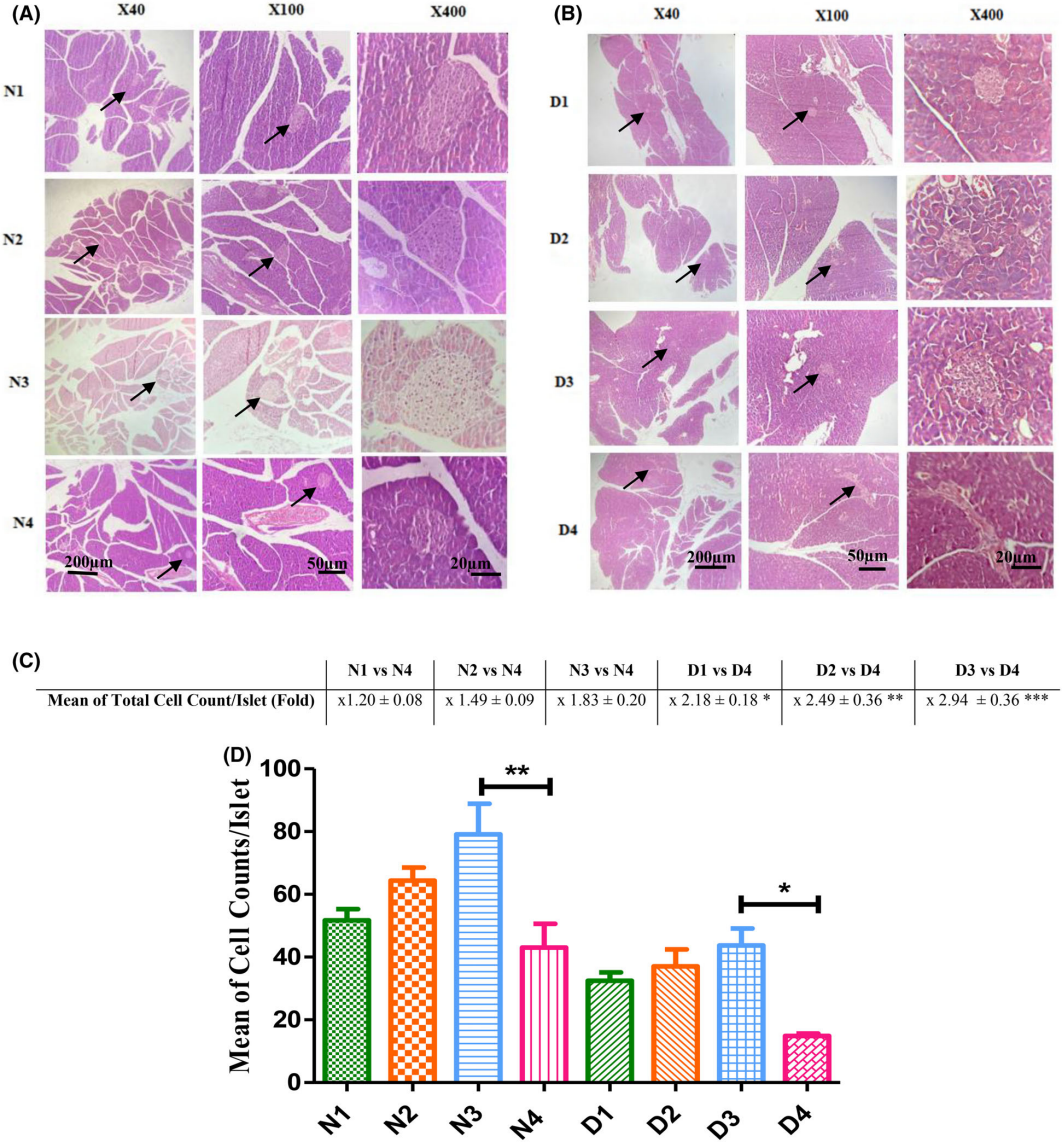
Hematoxylin and eosin staining of pancreatic sections at 40×, 100× and 400× magnification. Microscopic images of pancreatic tissue sections of treated‐normal and its control (A), treated‐diabetic and its control (B) are shown. Quantitative fold comparison of cell numbers per islet in each normal (C) and diabetic (D) treated groups versus their matched controls are shown. ***P < 0.001, **P < 0.01, *P < 0.05 (ANOVA). All data are depicted as the mean ± SEM (*n* = 5). Scale bars = 20, 50 and 200 μm (as indicated). Animal group classifications are as shown in the legend to Fig. 2.

As shown in Fig. 3A,B, GH, Mag and their combination improved the size of islets in both normal and diabetic mice. In Fig. 3C, the fold increase of cell count per islet is shown for normal and diabetic treated groups versus their matched controls. The fold increase is significant only in treated diabetic mice compared to untreated diabetic ones (D4). Also, and based on comparative analysis presented in Fig. 3D, only in the diabetic and normal mice treated with Mag and then growth hormone (D3 and N3), the average number of cells per islet increased by 2.94‐ and 1.84‐fold, respectively, relative to their relevant controls (D4 and N4). For groups treated solely with Mag or GH, the increase was not significant compared to their relevant controls (Fig. 3D).

### Effects of GH, Mag and Mag plus GH on key signaling elements

**Fig. 4:**
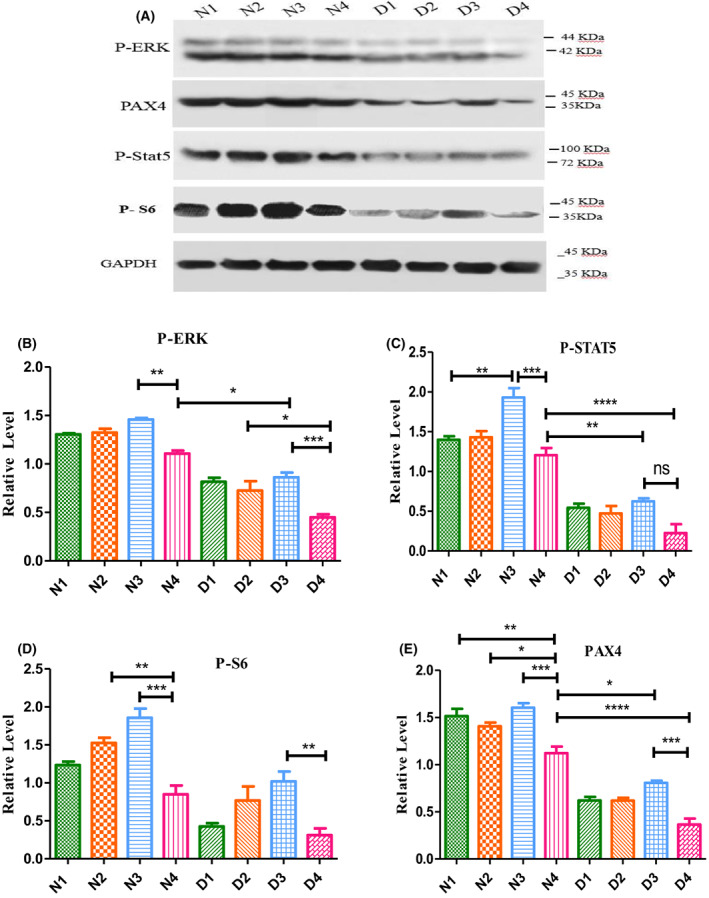
Western blot analyses of P‐ERK, P‐STAT5, P‐S6 and PAX4 in normal‐ and diabetic‐treated mice relative to their relevant controls. A prototype image of the immunoblot of pancreatic P‐ERK, P‐STAT5, P‐S6, PAX4 and GAPDH is shown in (A). Comparative immunoblot analysis for pancreatic P‐ERK (B), P‐STAT5 (C), P‐S6 (D) and PAX4 (E) is shown. ****P < 0.0001, ***P < 0.001 **P < 0.01, *P < 0.05; ns, no significant (ANOVA). All data are depicted as the mean ± SEM (*n* = 3–5). Analyses of immunoblot results were achieved using fiji‐java6 (https://downloads.imagej.net/fiji/Life-Line/fiji-java6-20170530.zip) and prism, version 5. Animal group classifications are as shown in the legend to Fig. 2.

Regarding the improvements in the mice pancreatic islet sizes and the number of cells per islet upon treatment with GH, Mag or their combination (Fig. 3), we evaluated the expression of ERK, STAT5, S6 and PAX4 signals following the Mag/GH treatments. In normal mice, there was no significant difference in P‐ERK levels for the N1 and N2 groups. However, the P‐ERK level in the N3 group significantly increased by approximately 1.31‐fold relative to the N4 group. With almost the same pattern, the extent of activation of ERK was highest for the D3 groups by 1.91‐fold relative to the relevant controls (Fig. 4A,B). Inspection of the western blot images (Fig. 4A) indicates that phosphorylation of STAT5 for all diabetic groups is significantly suppressed relative to all normal groups. However, the extent of P‐STAT5 is potentiated by almost 1.61‐ and 2.76‐fold upon co‐treatment with GH and Mag for the N3 and D3 groups relative to their relevant controls (N4 and D4), respectively (Fig. 4A,C). Similarly, the expression of P‐S6 significantly increased by 2.19‐fold for the N3 group relative to the N4 group, and D3 group 3.29‐fold relative to the D4 group (Fig. 4A,D). The expression level of PAX4, as the main marker for α to ß cell conversion, was also increased among the N1, N2 and N3 groups by almost 1.35‐, 1.25‐ and 1.43‐fold, respectively, whereas those of D1 and D2 groups were small and not significant. However, PAX4 expression increased by almost 2.21‐fold for the D3 group (Fig. 4A,E).

### Compensation of β cell mass among treated diabetic mice

**Fig. 5:**
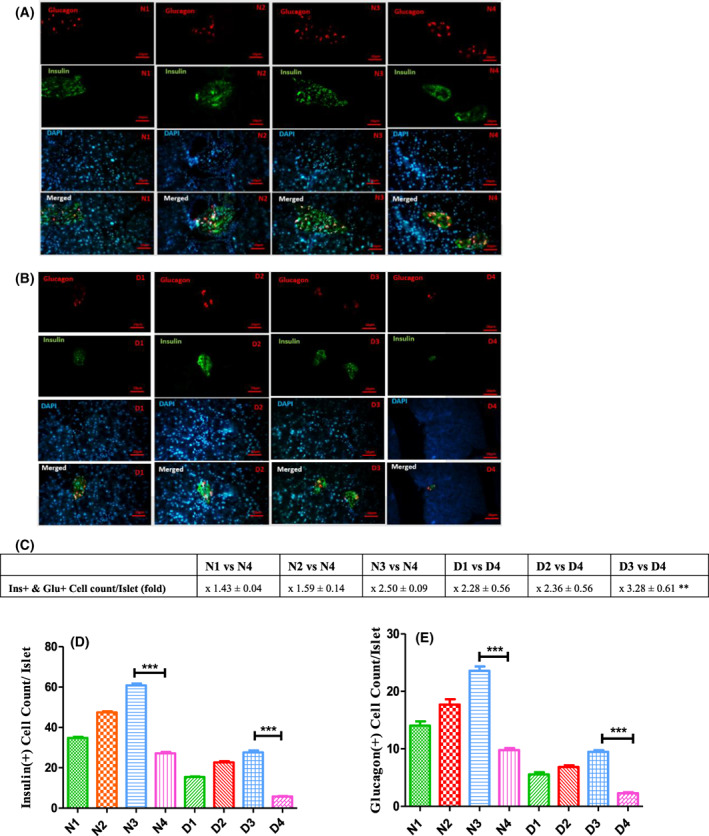
Magainin and GH induce insulin^+^ and glucagon^+^ cell regeneration in pancreatic islets. (A, B) Immunohistochemical staining performed on pancreas sections. (A) Normal treated/untreated. (C) Fold increase of insulin^+^/glucagon^+^, both cell count/pixel in normal‐ and diabetic‐treated mice versus their matched controls. (D, E) Respectively comparing the mean of insulin^+^ and glucagon^+^ cell count/islet of each group with results of other groups. *P < 0.05, **P < 0.01, ***P < 0.001, ****P < 0.0001 (one‐way ANOVA, *n* = 3). All data are depicted as the mean ± SEM in (C) to (E). Scale bar = 20 μm. Animal group classifications are as shown in the legend to Fig. 2.

Inspection of Fig. 5 supports α and ß‐like cell mass augmentation upon each treatment among the N and D groups, with higher variation among the N_3_ and D_3_ groups relative to their relevant controls. This is evident from Fig. 5A,B. The fold of cell/pixel counts of each group versus their controls presented in Fig. 5C. Comparative results of the mean cell count per islet are presented in Fig. 5D,E. As shown in Fig. 5C, the insulin^+^ and glucagon^+^ cells, among all normal‐treated groups, increased by almost 1.05‐ to 2.5‐fold relative to those of the N_4_ group. The regeneration of α and ß‐like cells among all diabetic‐treated groups is even more pronounced (between 2.19‐ and 4.75‐fold) relative to those of D4 mice. Also, Ins + and Glu + Cell count/Islet fold (bihormonal cells), as a marker of α to ß cell trans‐differentiation, significantly increased in the D3 compared to D4 groups.

**Fig. 6:**
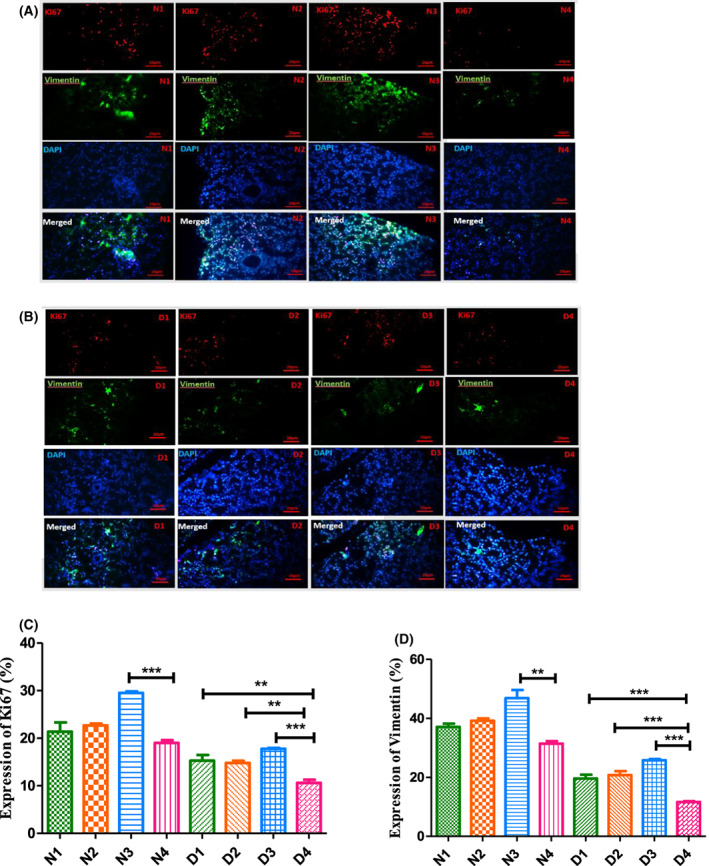
Magainin and GH induced ki67^+^ and vimentin^+^ cells of pancreas. (A, B) Immunohistochemical staining performed on pancreas sections. (A) Normal treated/untreated mice. (B) Diabetic treated/untreated. (C, D) Comparing the percentage of ki67^+^ and vimentin^+^ pixels of each group with those of other groups. **P < 0.01, ***P < 0.001 (one‐way ANOVA, *n* = 3). All data are depicted as the mean ± SEM in (C) and (D). Scale bar = 20 μm. Animal group classifications are as shown in the legend to Fig. 2.

Similarly, for immunohistochemical evaluation in normal and diabetic mice, Fig. 6A,B, shows that the number of Ki 67^+^ pixel count increased by 1.55‐fold (N_3_) and 1.67‐fold (D_3_) and that of vimentin^+^ pixels rose by 1.49‐fold (N_3_) and 2.21‐fold (D_3_). These data clearly support a higher proliferation and differentiation of epithelial to mesenchymal cells. It is clear that the combination treatments (N_3_ and D_3_) provide better results compared to the N_1_, N_2_, D_1_ and D_2_ groups (Fig. 6C,D).

### Suppression of the immune T and B cell numbers following treatments

**Fig. 7:**
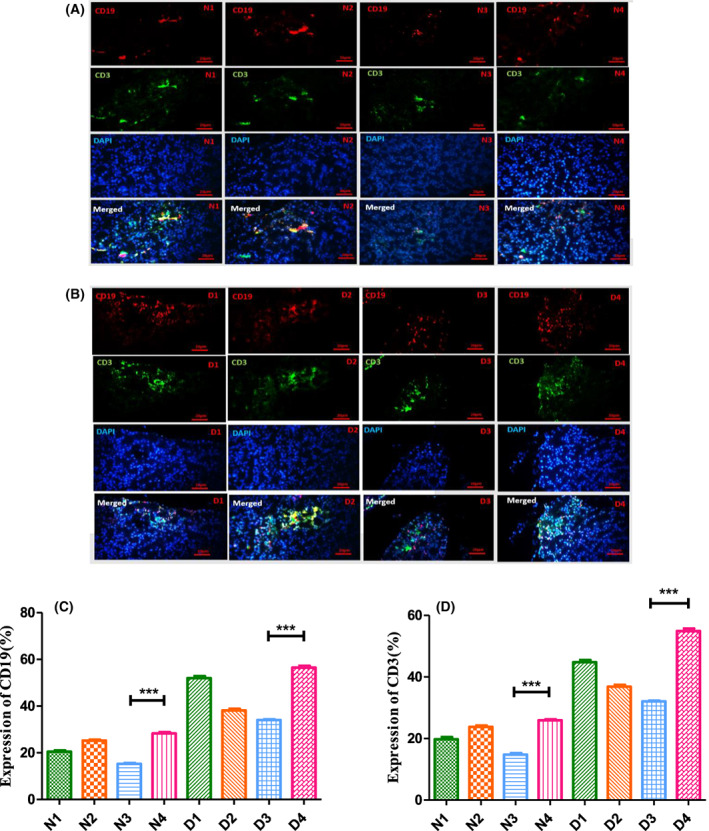
Magainin and GH induce reduction of CD19^+^ and CD3^+^ cells in pancreas. (A, B) Immunohistochemical staining performed on pancreas sections. (A) Normal treated/untreated mice. (B) Diabetic treated/untreated mice. (C, D) Alteration of CD19^+^ and CD3^+^ pixels in normal and diabetic treated mice and their matched controls. ***P < 0.001 (one‐way ANOVA, *n* = 3). All data depicted as mean ± SEM in (C) and (D). Scale bar = 20 μm. Animal group classifications are as shown in the legend to Fig. 2.

The general cell surface antigens of T and B lymphocytes are known as CD3^+^ and CD19^+^, respectively. Figure 7 indicates that the diabetic D4 mice have the most CD3^+^ and CD19^+^ pixels. The results summarized in Fig. 7C,D confirm that combination treatment with Mag and then GH is more effective with respect to decreasing the number of T and B cells. Qualitative comparison of Figs 6B and 7A demonstrates that the amount of CD3^+^ and CD19^+^ pixels effectively decreased by 60% and 58% in D3 mice and also by 57% and 54% among N3 mice, respectively (Fig. 7C,D).

## Discussion

Despite numerous reports on GH functions, there are a few studies on the anti‐diabetic effect of magainins. Magainin‐AM1 and ‐AM2, via stimulating GLP‐1 release from GluTag cells [9], can improve β cell mass maintenance by prohibiting apoptosis and enhancing cell proliferation [18, 19]. In addition, Mag‐AM2 enhances insulin release from mouse β cells via depolarization of the cell membranes and enhancing intracellular calcium [6]. Magainin‐II is also capable of increasing mice hypothalamic GABA content via direct activation of GAD, which produces GABA from l‐glutamate [11]. Recently, it was shown that GABA administration to STZ‐treated diabetic mice has led to regeneration of α and ß‐like cells and α cell conversion to ß‐like functional cells among mice pancreatic islets [12]. Furthermore, another recent study [20] indicates that, in a model of multiple low dose STZ‐induced diabetes, GLP‐1 (based on a meta‐analysis), insulin and GABA‐induced α and ß cell proliferation and α‐ to ß‐cell trans‐differentiation, whereas the proliferation ratio of ß cell/α cell increased only in GABA treated models. Moreover, α‐cell apoptosis was higher than that of ß cells in all groups. Our present data, in addition to being in line with the data in the literature, clearly confirms that endogenous induction of GABA [12] and GLP‐1 (Fig. S1) brings about the same results as claimed previously [12, 20].

Detailed inspection of the data in Fig. 4C indicates that fold increase of insulin^+^ cells is lower than the fold increase of glucagon^+^ cells among all normal‐treated groups. Inspection of the same counts among the diabetes‐treated groups, however, indicates that the fold increase of insulin^+^ cells is higher than the fold increase of glucagon^+^ cells. These data indicate that Mag, and/or its combination treatment, enhances the production of α cells among healthy animals but does not convert all of them to ß‐like cells. In other words, the requisite for conversion of α to ß‐like cells is the loss or ablation of ß cells, which occurs among the STZ‐treated animals, as also claimed previously [12].

Production of a higher than normal number of α and/or ß‐like cells might activate the immune system, which possesses the memory of ß cell antigens. To check this possibility, we evaluated the total population of T and B cells among all N and D subgroups. The results (Fig. 7) indicated that the mean pixel counts of CD19^+^ and CD3^+^among all treated groups were lower than their relevant control groups (N_4_ and D_4_). These data clearly indicate that not only is the immune system is not ignited to the level of detection following Mag, and/or Mag^+^ GH treatments, but also it is partially suppressed among the STZ‐induced diabetic mice.

In conclusion, the present *in vivo* data provide evidence regarding the possibility of the pharmaceutical induction of α/β cell production and their trans‐differentiation to functional β‐ like cells without igniting the immune system via administration of Mag and, to a better extent, via combined administration of Mag and GH. These findings could finally pave the path toward an alternative insulin replacement therapy pending scientific maturation of the subject at the clinical level.

## Conflicts of interest

The authors declare that they have no conflicts of interest.

## Author contributions

AM and RY contributed to the study conception and design. AM was responsible for material preparation and data collection. AM and RY conducted the analyses. AM wrote the first draft of the manuscript, with major editing by RY.

## Supporting information


**Fig. S1.** The Mag and GH signaling pathways affecting the β‐cell fate. ARX, aristaless related homeobox; ERK, extracellular signal regulated kinase; FOXO1, forkhead box protein O1; GABA, gamma‐aminobutyric acid; GLP‐1, glucagon‐like peptide 1; PAX4, paired box 4; Pdx1, pancreatic and duodenal homeobox 1; PRLR, prolactin receptor; STAT, signal transducer and activator of transcription.

## Data Availability

The data that support the findings of this study are available from the corresponding author upon reasonable request.
