# Supplementary material for: Beta cell regeneration upon magainin and growth hormone treatment as a possible alternative to insulin therapy
Source: FEBS Open Bio. 2023 Jan 25;13(3):447–58. doi: 10.1002/2211-5463.13556 (PMC9989927; doi:10.1002/2211-5463.13556)
Supplement: Supplementary file 1 — Fig. S1. The Mag and GH signaling pathways affecting the β‐cell fate. ARX, aristaless related homeobox; ERK, extracellular signal regulated kinase; FOXO1, forkhead box protein O1; GABA, gamma‐aminobutyric acid; GLP‐1, glucagon‐like peptide 1; PAX4, paired box 4; Pdx1, pancreatic and duodenal homeobox 1; PRLR, prolactin receptor; STAT, signal transducer and activator of transcription. [file FEB4-13-447-s001.docx]

**Supplementary Materials**

**Beta cell regeneration upon Magainin and growth hormone treatments: A promising alternative approach for insulin therapy**

Azam Moosavi and Razieh Yazdanparast*

Institute of Biochemistry and Biophysics, University of Tehran, Tehran, Iran

*Corresponding author: R. Yazdanparast

Institute of Biochemistry and Biophysics

University of Tehran, Tehran, Iran

e-mail [ryazdan@ut.ac.ir](mailto:ryazdan@ut.ac.ir)

Tel +98-021-66956976

iPhon 0912 137 2922


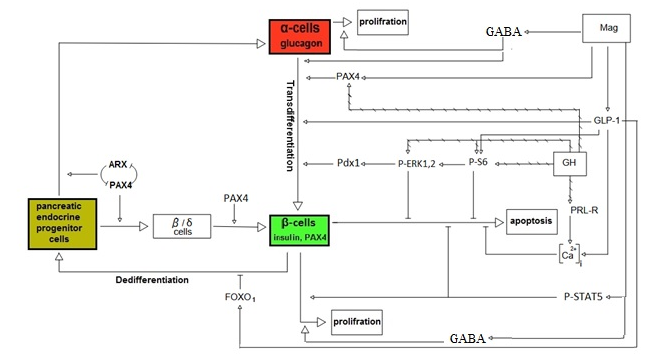


**Fig. S1**-The Mag and GH signaling pathways affecting the β- cell fate. Extracellular Signal regulated kinase (ERK), Forkhead box protein O1 (FOXO1), Glucagon-like peptide1 (GLP-1), Paired box 4 (PAX 4) and Pancreatic and duodenal homeobox 1(Pdx 1).
